# Misinformation versus Facts: Understanding the Influence of News regarding COVID-19 Vaccines on Vaccine Uptake

**DOI:** 10.34133/2022/9858292

**Published:** 2022-03-12

**Authors:** Hanjia Lyu, Zihe Zheng, Jiebo Luo

**Affiliations:** ^1^Department of Computer Science, University of Rochester, Rochester, USA; ^2^Goergen Institute for Data Science, University of Rochester, Rochester, USA

## Abstract

*Background*. There is a lot of fact-based information and misinformation in the online discourses and discussions about the COVID-19 vaccines.*Method*. Using a sample of nearly four million geotagged English tweets and the data from the CDC COVID Data Tracker, we conducted the Fama-MacBeth regression with the Newey-West adjustment to understand the influence of both misinformation and fact-based news on Twitter on the COVID-19 vaccine uptake in the US from April 19 when US adults were vaccine eligible to June 30, 2021, after controlling state-level factors such as demographics, education, and the pandemic severity. We identified the tweets related to either misinformation or fact-based news by analyzing the URLs.*Results*. One percent increase in fact-related Twitter users is associated with an approximately 0.87 decrease (B=−0.87, SE=0.25, and p<.001) in the number of daily new vaccinated people per hundred. No significant relationship was found between the percentage of fake-news-related users and the vaccination rate.*Conclusion*. The negative association between the percentage of fact-related users and the vaccination rate might be due to a combination of a larger user-level influence and the negative impact of online social endorsement on vaccination intent.

## 1. Introduction

Many people read news on social media today, yet the veracity of the news is not guaranteed. Waszak et al. studied the top shared health web links on Polish social media platform and found that 40% of the most frequently shared links contain fake news [1]. Fake news regarding the COVID-19 pandemic is particularly concerning. By identifying and analyzing 1,225 pieces of COVID-19 fake news, Naeem et al. concluded that fake news is pervasive on social media, putting public health at risk [2]. Among these health-related fake news, vaccine-related news [3] has the most fallacious content [1]. A recent study showed that misinformation induced a decline in intent of 6.2% in the UK and 6.4% in the USA among those who previously intended to take the vaccine [4]. To support the COVID-19 vaccination, Rzymski et al. [4] suggested to track and tackle emerging and circulating fake news. Montagni et al. [5] argued to increase people’s ability to detect fake news. Additionally, collaboration with the media and other organizations should be used, given that citizens do not support the involvement of government authorities in the direct control of news [6]. By studying the antivaccination sentiment on Facebook, Hoffman et al. concluded that it would be valuable for health professionals to deliver targeted information to different subgroups of individuals through social networks [7]. In this study, we intended to examine the scale and scope of the influence of misinformation and fact-based news about COVID-19 vaccines on social media platforms on the vaccine uptake. To summarize, this work (1) quantitatively analyzed the effect of fake news and fact-based news on the vaccine uptake in the US using the Fama-MacBeth regression with the Newey-West adjustment and (2) compared the user characteristics of the fact-related and fake-news-related users. *Seemingly counter-intuitive*, the percentage of fact-related users is significantly negatively associated with the vaccination rate while no significant correlation is found between the percentage of fake-news-related users and the vaccination rate. The fact-related users have relatively more social capitals than the fake-news-related users. Most of the frequent keywords in the user descriptions of the fake-news-related users are political.

## 2. Material and Method

### 2.1. Datasets

#### 2.1.1. Twitter Data

We used the Twitter API (https://www.tweepy.org/, Accessed June 9, 2021) to collect the related tweets that were publicly available. More specifically, the Twitter streaming API was used. The search keywords and hashtags are COVID-19 vaccine-related or vaccine-related, including “vaccine,” “vaccinated,” “immunization,” “covidvaccine,” and “#vaccine” (the capitalization of nonhastag keywords does not matter in the Tweepy query). Slang and misspellings of the related keywords were also included which are composed of “vacinne,” “vacine,” “antivax,” and “anti vax.” The tweets that were only related to other vaccine topics like MMR, autism, HPV, tuberculosis, tetanus, hepatitis B, flu shot, or flu vaccine were removed using a keyword search. Moreover, since this study focused on the tweets posted by the US Twitter users, we used the geolocation disclosed in the users’ profiles to filter out the tweets of non-US users. Similar to Lyu et al. [8], the locations with noise were excluded. Nearly four million geotagged tweets as well as the retweets posted from April 19, 2021, to June 30, 2021, were collected. The average number of geotagged tweets per state is 79,894 (Min=2,796,Max=715,871, and SD=119,637).

#### 2.1.2. CDC COVID-19 Data

The daily state-level number of people with at least one dose, confirmed cases, and deaths per hundred were extracted from the CDC COVID Data Tracker [9].

#### 2.1.3. Census Data

Multiple factors including demographics, socioeconomic status, political affiliation, and population density have been found to be related with people’s intent to accept a COVID-19 vaccine [10,11]. These were considered as control variables in our study. From the latest American Community Survey 5-Year Data (2015-2019) [12], we collected (1) the percentage of male persons, (2) the percentage of persons aged 65 years and over, (3) the percentage of Black or African American alone, (4) the percentage of Asian alone, (5) the percentage of Hispanic or Latino, (6) the percentage of other, (7) the percentage of persons aged 25 years and over with a bachelor’s degree or higher, (8) the percentage of persons in the labor force (16 years and over), (9) per capita income in the past 12 months (in 2019 dollars), and (10) the percentage of urban population. For these control variables, the state-level numbers were extracted.

#### 2.1.4. 2020 National Popular Vote Data

The results of the 2020 national popular vote [13] were used to estimate the political affiliation of individual states. Since the sums of the shares of Biden and the shares of Trump are almost equal to 100%, we only selected the shares of Biden. To keep the consistency among the variables, the state-level shares were chosen.

### 2.2. Tweet Classification

On the one hand, automated fake news detection methods have been proposed by multiple studies. To characterize fake news, Zhou and Zafarani represented the spread network in different levels and confirmed that the network of misinformation is more-spread, farther in distance, and denser [14]. Horne and Adali found that fake news has longer titles, uses simpler sentences, and is more similar to satire compared to real news [15]. Sentiment of the content was also proven to be an important feature used to detect fake news [16]. Jin et al. [17] proposed a recurrent neural network with an attention mechanism to fuse multimodal features for effective rumor detection. On the other hand, researchers also relied on fact-checking groups to detect misinformation [18]. Compared to the automated fake news detection methods, this kind of approach has a higher true positive rate and a lower false positive rate. A relatively low recall rate could be a disadvantage. However, a previous study has shown that it still enabled researchers to reveal important patterns and insights [18]. To have a better and more precise understanding of the influence and importance, we detected misinformation using the second type of approach. 

In particular, following the method of Bovet and Makse [18], we attempted to classify the tweets into (1) fake-news-related, (2) fact-related, and (3) others, by examining the URLs (if any) of the tweets. More specifically, if the URL’s domain name was judged on the basis of the opinion of communications scholar to be related to the websites containing fake news, conspiracy theories, unreliable contents, or extremely biased news, the tweets that were associated with (i.e., contained/retweeted/quoted) this URL were classified as fake-news-related. It is noteworthy that not only the websites containing fake news, but also the ones containing conspiracy theories, unreliable contents, or extremely biased news, were included in this group. The websites containing extremely biased news are sources “that come from a particular point of view and may rely on propaganda, decontextualized information, and opinions distorted as facts by http://www.opensources.co” [18]. For simplicity, we refer to this group of websites containing fake news, conspiracy theories, unreliable contents, or extremely biased news as fake-news-related. 

If the URL’s domain name was judged to be related to the websites that were traditional, fact-based, news outlets, the tweets that were associated with this URL were classified as fact-related. If the tweets were not associated with any URLs or the URLs’ domain names were not identified as fake-news-related or fact-related, the tweets were classified as others. Most URLs were shortened. We used the Python Requests package to open the URLs and extracted the actual domain names from the complete URLs.

The curated list of fake-news-related websites, composed of 1,125 unique domain names, was built by the Columbia Journalism Review. (https://www.cjr.org/, Accessed January 31, 2022) They built the list by merging the major curated fake-news site lists provided by fact-checking groups like PolitiFact, FactCheck, OpenSources, and Snopes. The domain names that were assigned as fake, conspiracy, bias, and unreliable were included in our study. 

The curated list of fact-related websites, composed of 77 unique domain names, was reported by Bovet and Makse [18]. They identified the most important traditional news outlets by manually inspecting the list of top 250 URLs’ domain names. 

Using this approach, we assumed that the Twitter users did not post a tweet containing a link to fake news outlets or fact-based news outlets just to indicate whether or not they thought the content was fact or fake. Example tweets are as follows:

(i) This article in [fake-news-related URL] is apparently fake/a fact
(ii) This article in [fact-related URL] is apparently fake/a fact

Instead, we assumed that the Twitter users shared a similar opinion with the content they posted. To verify the assumption and the robustness of our approach, we randomly sampled 100 unique tweets from both identified fake-news-related (fake news, conspiracy theories, unreliable contents, or extremely biased news) and fact-related tweets, respectively, and inspected whether or not they met our assumptions. After manually reading the sampled tweets, we found all of them met our assumptions. In fact, the majority of the contents are just a short sentence that summarizes the content to which the URL links.

### 2.3. Preprocessing

For the daily state-level number of people with at least one dose, confirmed cases, deaths per hundred were transformed using a two-step procedure. First, we calculated the lag-1 differences of these three variables. Next, we smoothed the data using a simple moving average. According to the CDC vaccination data [9], there is a seasonal pattern inside the number of daily new vaccinated people (i.e., the lag-1 difference). The number normally reaches the highest on Thursdays or Fridays and approaches the lowest on weekends. Therefore, we applied a 7-day moving average to the vaccination data. To maintain the consistency, the lag-1 differences of confirmed cases and deaths were processed in the same way. 

As for the Twitter data, since Twitter users could post tweets repeatedly, the series of (1) the percentage of unique Twitter users who posted fake-news-related tweets and (2) the percentage of unique Twitter users who posted fact-related tweets were only processed with a 7-day moving average.

### 2.4. Fama-MacBeth Regression

In our study, we attempted to analyze five time series data, but most of them are nonstationary. For example, the time series of the vaccination data show a declining trend during our study period. Noticeably, the vaccination data, at this stage, has already been transformed into the lag-1 difference. To avoid the spurious regression problem, which might lead to a incorrectly estimated linear relationship between nonstationary time series variables [19], we conducted the Fama-MacBeth regression [20] with the Newey-West adjustment [21], which has also been applied in several previous studies to address the time effect in areas such as finance [22], public health, and epidemiology [23]. The optimal number of lags was selected automatically using a nonparametric method [24]. Apart from the time series data, we added control variables from the aforementioned data sources including the Census data and the 2020 National Popular Vote data. Table 1 summarizes the dependent, independent, and control variables. 

**Table 1:** Variables of interest.

	Variables
Dependent	Daily new vaccinated people per hundred (7-day average)
Independent	(1) Percentage of fake-news-related (fake news, conspiracy theories, unreliable contents or extremely biased news) tweets (7-day average)
	(2) Percentage of fact-related tweets (7-day average)
Control	(1) Percentage of male persons
	(2) Percentage of persons aged 65 years and over
	(3) Percentage of Black or African American alone
	(4) Percentage of Asian alone
	(5) Percentage of Hispanic or Latino
	(6) Percentage of other
	(7) Percentage of persons aged 25 years and over with a bachelor’s degree or higher
	(8) Percentage of persons in the labor force (16 years and over)
	(9) Per capita income in the past 12 months (in 2019 dollars)
	(10) Percentage of urban population
	(11) Daily new cases per hundred (7-day average)
	(12) Daily new deaths per hundred (7-day average)

## 3. Results

Using the aforementioned URL-based tweet classification method, we detected 26,998 fake-news-related (fake news, conspiracy theories, unreliable contents, or extremely biased news) and 456,061 fact-related tweets. There were 10,925 unique Twitter users who were associated with fake news, while 159,283 were associated with fact-based news. Interestingly, 6,839 were associated with both fake news and fact-based news, which accounted for 62.6% and 4.3% of the fake-news-related users and fact-related users, respectively. This suggested that people who were associated with fake news were more likely to be associated with fact-based news, but not the other way around.

**Figure 1:**
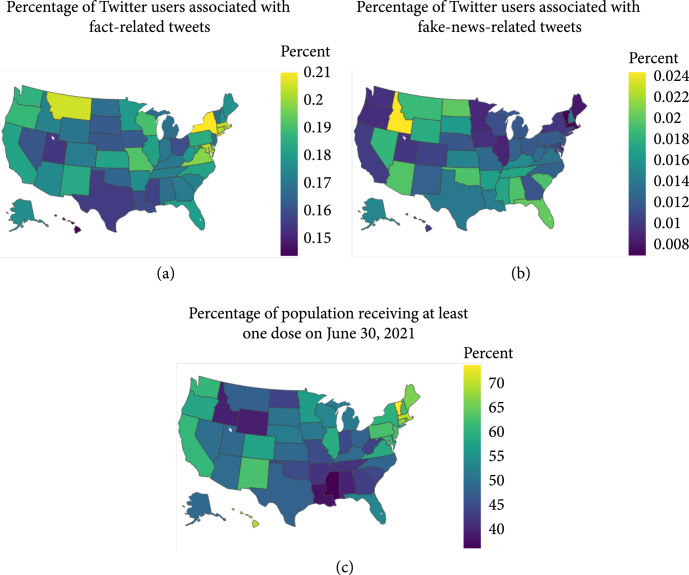
State-level visualization of (a) fact-related and (b) fake-news-related (fake news, conspiracy theories, unreliable contents, or extremely biased news) tweets and (c) COVID-19 vaccination rates. The percentages of unique Twitter users associated with fact-related and fake-news-related tweets were used for (a, b). Percentages of population receiving at least one dose of COVID-19 vaccine as of June 30, 2021, were used for (c).

The state-level percentages of fake-news-related and fact-related Twitter users are presented in Figures 1(a) and 1(b). Overall, there is clear segregation between the states with more users associated with fact-related tweets and the states with more users associated with fake-news-related tweets. They tend to be geographically close to each other within the same group. For instance, there are more users associated with fact-related tweets in New York, Connecticut, and Massachusetts. Higher percentages of users associated with fake-news-related tweets are observed in the Southeast of the US.

We conducted the Fama-MacBeth regression with the Newey-West adjustment of the 7-day average number of daily new vaccinated people per hundred, on the 7-day average percentages of unique fake-news-related (fake news, conspiracy theories, unreliable contents, or extremely biased news) and fact-related Twitter users, during the period when all US adults were eligible for COVID-19 vaccines, while controlling other factors. The CDC vaccination data might not reflect the intention to receive vaccination when the vaccines were not available for all the US adults. We thus set the start date of the study period to be April 19, 2021, because, according to the Reuters (https://www.reuters.com/article/us-health-coronavirus-usa/all-american-adults-to-be-eligible-for-covid-19-vaccine-by-april-19-biden-idUKKBN2BT1IF?edition-redirect=uk, Accessed June 9, 2021), President Joe Biden moved up the COVID-19 vaccine eligibility target for all American adults to April 19. The regression was conducted using approximately 3-month data (from April 19, 2021, to June 30, 2021).

Figure 1(c) shows the state-level vaccination rates as of June 30, 2021. Compared to Figures 1(a) and 1(b), we found that some of the states with relatively lower vaccination rates tend to have both higher rates of fake-news-related and fact-related users, perhaps an indication of higher disagreement (e.g., Montana, Idaho, and Wyoming). Table 2 summarizes the results of the Fama-MacBeth regression, which suggests a significant effect of the fact-based news on the vaccination rates. The percentage of fact-related Twitter users is negatively associated with the vaccination rates: one percent increase in fact-related Twitter users is associated with an approximately 0.87 decrease (B=−0.87, SE=0.25, and p<.001) in the number of daily new vaccinated people per hundred. This is *consistent* with the findings of Loomba et al. [25], where they conducted a questionnaire-based randomized controlled trial to quantify the effects of exposure to online misinformation around COVID-19 vaccines over vaccination intent. The percentages of people holding negative opinions (“leaning no” and “definitely not”) about COVID-19 vaccines indeed increase after exposure to factually correct information. It is also noteworthy that the increase is consistent across all four experiment settings in their study. However, what is inconsistent between our findings and theirs is the effect of misinformation. They found the exposure to misinformation induces a decline in vaccination intent, while, as shown in Table 2, we did not find significant relationship between the percentage of fake-news-related users and the vaccination rate.

**Table 2:** The results of the Fama-MacBeth regression with the Newey-West adjustment.

Variable	B	SE	t-stat	p value
Fake news	-0.34	0.59	-0.58	0.566
Fact-based news	-0.96 ^∗∗∗^	0.27	-3.53	<.001
Male	-1.23	0.95	-1.29	0.201
65 years and over	0.15	0.12	1.28	0.206
Black	-0.23 ^∗∗∗^	0.06	-3.84	<.001
Asian	0.39 ^∗^	0.19	2.01	0.048
Hispanic	-0.11	0.10	-1.17	0.244
Other	-0.07	0.15	-0.50	0.616
Bachelor	5.56 e-3 ^∗∗∗^	1.38 e-3	4.04	<.001
Labor	-4.24 e-3 ^∗∗∗^	8.61 e-4	-4.92	<.001
Income	-3.92 e-6 ^∗∗^	1.21 e-6	-3.24	0.002
Urban	4.72 e-4 ^∗^	2.27e-4	2.08	0.041
Confirmed cases	0.41	0.41	0.99	0.325
Deaths	-28.96	24.27	-1.19	0.237
Biden shares	5.31 e-3 ^∗∗∗^	1.09 e-3	4.90	<.001
const	0.86	0.44	1.94	0.056

Note. ^∗^*p*<0.05. ^∗∗^*p*<0.01. ^∗∗∗^*p*<0.001.

For the control variables, on the one hand, some of the variables are significantly associated with the vaccination rate. As for the race and ethnicity, the percentage of Asian alone (B=0.39, SE=0.19, and p<.05) is positively associated with the vaccination rate, while the percentage of Black or African American alone is negatively associated (B=−0.23, SE=0.06, and p<.001). With respect to the educational level, one percent increase in the percentage of persons aged 25 years and over with a bachelor’s degree or higher is associated with an approximately 5.56 e-3 increase (B=5.56e−3, SE=1.38e−3, and p<.001) in daily new vaccinated people per hundred. Socioeconomically, per capita income (in 2019 dollars) is negatively associated with the vaccination rate (B=−3.92e−6, SE=1.12e−6, andp<.001). One percent increase in the percentage of urban population is associated with a 4.72 e-4 increase (B=4.72e−4, SE=2.27e−4, andp<.05) in the vaccination rate. The vaccination rates are higher among the states with a relatively higher percentage of people voting for Biden (B=5.31e−3, SE=1.09e−3, and p<.001). On the other hand, gender, Hispanic or Latino, other, the numbers of daily new confirmed cases, and deaths are not found to be significantly associated with the vaccination rate.

**Figure 2:**
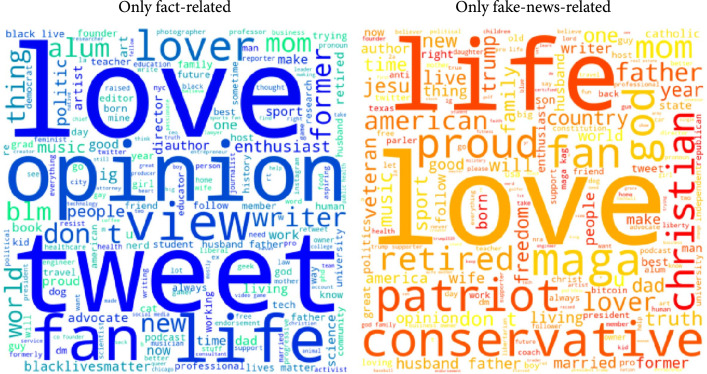
User descriptions of the users who have either posted fact-related or fake-news-related tweets but not both.

To better understand the discrepancy in the effects of misinformation (fake news, conspiracy theories, unreliable contents, or extremely biased news) on vaccination intent between our findings and the findings of Loomba et al. [25], we dived deep into the user characteristics by comparing the social capitals (e.g., the number of followers) of two groups of Twitter users—one group composed of the users who have posted fake-news-related tweets but have not posted fact-related tweets and the other composed of the users who have posted fact-related tweets but have not posted fake-news-related tweets. Since the social capitals are not normally distributed, we performed the Mann-Whitney rank test with the Bonferroni correction on the numbers of followers, friends, statuses, favorites, and listed memberships. There is significant evidence (p<.05) to conclude that the social capitals of these two groups of users are different. Specifically, the users who have posted fact-related tweets but have not posted fake-news-related tweets have more followers, friends, statuses, listed memberships, and give more favorites (i.e., likes). Moreover, by performing the proportion z test over the percentages of verified users between these two groups, we found there are significantly more verified users among the fact-related Twitter users (p<.05). We further plotted the word clouds of the user descriptions of these two groups in Figure 2. The size of the word is proportional to its frequency. Apart from “love” and “life” which appear in both groups, a clear difference can be observed between the other keywords of the user descriptions. Political keywords such as “maga,” “conservative,” and “Trump” are in the user descriptions of the users who posted fake-news-related tweets. However, there are fewer political keywords in the user descriptions of the users who posted fact-related tweets, although there are “blm” and “blacklivesmatter.” 

## 4. Discussions

We identified a significant negative correlation between the percentage of the US Twitter users who were associated with fact-based news and the US COVID-19 vaccination rates during the period when all US adults were eligible for the COVID-19 vaccines. We found no significant effects of misinformation (fake news, conspiracy theories, unreliable contents, or extremely biased news) on the vaccination rates. The negative relationship between the fact-based news and the vaccination rate is *consistent* with the questionnaire-based randomized control trials conducted by Loomba et al. [25]. However, we found discrepancy in the effects of misinformation. As acknowledged by Loomba et al. [25], their study “does not replicate a real-world social media platform environment where information exposure is a complex combination of what is shown to a person by the platform’s algorithms and what is shared by their friends or followers [26].” By comparing the user characteristics of the fact-related and fake-news-related users, we found significant evidence that the fact-related users tend to have greater online influence as they have more followers, friends, statuses, listed memberships, and give more favorites (i.e., likes) and are more likely to be verified users. We further qualitatively compared the words extracted from the user descriptions of these two groups of users and found clear differences. The fake-news-related users tend to have similar user profiles as more political keywords such as “maga,” “conservative,” and “Trump” were observed in the user descriptions. As a result, in our study, the number of detected fact-related users is almost 15 times of the number of detected fake-news-related users. These findings indicate a combination of a smaller online influence and a tendency for selective exposure to homogeneous opinions [27, 28] that may create echo chambers [29, 30].

At first glance, it might be counterintuitive that more fact-related news is associated with lower vaccination rate. However, this pattern was *consistently* found in both survey-based studies [25, 31] and our social media-based study. The reason could be that more fact-related news about the vaccines might raise not only more discussions but also more concerns. This nonpositive perception of the vaccines might induce a decline in the vaccination intent among the people who were hesitant. Chadwick et al. [31] conducted a survey-based study to explore the implications of online social endorsement for the COVID-19 vaccination program. They found the effects of online social endorsement are complex in terms of the people who consume them. The people who give less priority to active monitoring of news are more likely to be associated with discouragement of vaccination compared to the people who actively seek news. It is notable that the users we captured using our methods are the ones who posted tweets. Based on our results, the people who have posted fact-related tweets but have not posted fake-news-related tweets have a relatively larger audience. The people among the audience who do not post fact-related tweets can be considered as less active than the people who posted. Therefore, according to the findings of Chadwick et al. [31], these people might become more vaccine hesitant after consuming a growing amount of the news, which could be the reason of a negative association we found in our study. Future research can further explore this pattern by investigating the effects of online social endorsement on vaccination intent [31] using social media data in real-world environment.

With respect to the control variables, the patterns are *in line with* the ongoing vaccination trends [9]. In June 2021, the estimated percents of people 18 years and older in White alone, not Hispanic or Latino, Black or African American alone, and Asian alone were 66.8, 56.7, and 85.0, respectively. Our results also show a negative association between the percentages of Black or African American alone and a positive association between the percentages of Asian alone with the vaccination rate. No statistically significant relationship was found between the percentages of persons aged 65 and over, which is within our expectation, since this demographic group was among the first batches who were eligible for the COVID-19 vaccines in the US. By the time of our study period, over 78% of the people aged 65 years and over have already received at least one dose [9]. Echoed with Bertoncello et al. [32], the states with more people holding a bachelor’s degree or higher tend to have higher vaccination rates.

The findings of our study should be interpreted with caution as there are still limitations in terms of the representativeness of online behaviors and the potential biases in the type of people using Twitter. However, multiple previous studies have shown that this kind of online activities is representative of real-world patterns in many areas such as diet [33, 34] and public health [10, 35]. More importantly, as also shown in our study, social media-based studies to some extent overcome the challenges encountered using survey-based methods [25]. Ideally, a future research direction is to explore the combination of both survey-based and social media-based methods to improve robustness while addressing the drawbacks of both methods.

Moreover, this work employed a method to identify fake-news-related and fact-related tweets only using the URLs fact checked by human experts, which could potentially cause a sample bias since not all fake-news-related or fact-related tweets contain URLs. However, one of the advantages of this approach over other text-based machine learning or deep learning methods [17, 36, 37] is its high precision rate. Shahi and Nandini [38] presented a multilingual cross-domain dataset of 5,182 fact-checked news articles for COVID-19. This dataset was annotated manually. They used a BERT-based classification model [39] for fake/fact detection. The overall precision was only 0.78. Although this result was achieved without fine-tuning, it suggests that there are gaps in the precision rate between expert-labeled and machine-detected results. In the future, we intend to combine these methods to detect fake news more reliably. In addition, other advanced time series models can be explored to perform the regression analysis for spatial and temporal patterns.

## 5. Conclusion

In this study, we identified the tweets related to either misinformation (fake news, conspiracy theories, unreliable contents, or extremely biased news) or fact-based news posted from April 19, 2021, to June 30, 2021, on Twitter. After performing the Fama-MacBeth regression with Newey-West adjustment, we found the percentage of fact-related users is significantly associated with the vaccination rate. We did not find a significant relationship between the percentage of fake-news-related users and the vaccination rate. We further compared the user characteristics of the fact-related and fake-news-related users and found fact-related users have significantly more social capitals. The fake-news-related users are similar to each other in terms of social capitals as well as their user descriptions. Our findings are mostly consistent with the findings of previous survey-based studies. More importantly, we conducted our study by passively observing the social media data in an attempt to address the issue that previous survey-based studies did not replicate a real-world social media platform environment, enabling us to have a better understanding of the mechanism of the relationship between vaccine-related news and vaccination rates.

## Data Availability

The data used to support the findings of this study are available from the corresponding author upon request.
